# Experiences of primary caregivers of children with cerebral palsy across the trajectory of diagnoses in Ghana

**DOI:** 10.4102/ajod.v8i0.577

**Published:** 2019-09-25

**Authors:** Joana D.A. Kyeremateng, Anthony Edusei, Joslin A. Dogbe, Maxwell P. Opoku, William Nketsia, Charles Hammond, Sally A. Afriyie

**Affiliations:** 1 Centre for Disability and Rehabilitation Studies, Kwame Nkrumah University of Science and Technology, Kumasi, Ghana; 2 Department of Child Health, Kwame Nkrumah University of Science and Technology, Komfo Anokye Teaching Hospital, Kumasi, Ghana; 3 Faculty of Education, University of Tasmania, Hobart, Australia; 4 School of Education, Western Sydney University, Sydney, Australia

**Keywords:** cerebral palsy, caregiving, parents, culture, Ghana, children

## Abstract

**Background:**

Cerebral palsy (CP) is a non-progressive disorder of posture or movement caused by a lesion to the developing brain that results in functional limitations. The diagnosis of CP can vary from one child to another, causing family stress because of vague and unknown outcomes of the disorder. Although there are negative attitudes in Ghanaian societies towards primary caregivers and children with disabilities, fewer attempts have been made to understand their experiences.

**Objectives:**

The main aim of this study was to explore the experiences of primary caregivers across the trajectory of the diagnosis (before, during and after) of CP in the setting of a tertiary hospital.

**Method:**

Using Social Capital Theory as framework, 40 primary caregivers of children with CP, who were receiving treatment at a major referral hospital in Ghana, were interviewed about their experiences before, during and after diagnosis.

**Results:**

The results that emerged from the thematic analysis were discussed as follows: experiences before diagnosis, experiences during the diagnosis and experiences after the diagnosis. Particularly, participants discussed their inability to access essential services such as education for their children with CP.

**Conclusion:**

In light of systemic challenges faced by participants and their children with CP, the need for health policymakers to prioritise the public education about CP, promoting the well-being of caregivers and other implications of the study have been discussed.

## Introduction

Cerebral palsy (CP) is a non-progressive disorder of posture or movement caused by a lesion of the developing brain (Bulekbayeva et al. 2017; Oskoui et al. 2013). The gradual increase in the number of children diagnosed with CP has rekindled discussions on their welfare in societies (Sellier et al. 2015). For example, it was estimated the global prevalence of CP is two to three children per every 1000 births (Braun et al. 2015, 2016; Oskoui et al. 2013). In the Ghanaian context, although there are no official statistics on number of children with CP, Cerebral Palsy Africa (CPA) estimates that one child per 300 births has CP in Ghana. While the prevalence of CP in developed countries seemed to be declining (Braun et al. 2016; Sellier et al. 2015), in low-income countries the prevalence of the disorder remains high (Dan & Paneth 2017). Unfortunately, in low-income countries empirical evidence on the experiences of primary caregivers remains low (Dan & Paneth 2017). In societies such as Ghana, where current systems of care for children with disabilities are unavailable (e.g., Dogbe et al. 2019), primary caregivers are expected to raise their children and be part of the life of their children with CP. If parents are not healthy and received informal support from family members, they may be unable to provide for themselves, their child and the rest of their family with the best possible care. While there is no cure available for CP, if appropriate healthcare is available, children affected by CP without significant co-morbidities may have normal-to-near-normal life expectancies. In this study, we explored the perspectives of primary caregivers raising their children with CP in Ghana.

Cerebral palsy is a developmental disability that could impact on the life of growing children. The main feature of CP is impaired motor function that has impact on mobility of children (Braun et al. 2016; Sellier et al. 2015). In addition, some children with CP may have co-morbid conditions such as living with intellectual disability (Christensen et al. 2013). This could adversely impact on their daily living experiences and limitations with self-care functions such as feeding, dressing, bathing and mobility (Galpin et al. 2018; Rajan & John 2017). These limitations can result in the need for long-term care, far exceeding the usual needs of typical developing children. This means that primary caregivers of children with CP acquire the informal work of caregiving, with indefinite ending. In effect, CP does not only affect the children alone but rather may impact on their primary caregivers too (Majnemer et al. 2012). Therefore, there is the need for systems to be concerned about the well-being of not only the child with CP but also primary caregivers who are expected to provide children with ‘unending’ support services.

In Ghana, there is traditional interpretation given to people with disability that impacts their participation in societies. Disability is perceived as stigmatising condition, and giving birth to children with disabilities is linked to the work of supernatural forces (Agyei-Okyere et al. 2019; Kassah, Kassah & Agbota 2018; Opoku et al. 2017a, 2019). For instance, the birth of children with disabilities is interpreted as punishment from gods, for sins committed by a member of family (Opoku et al. 2017b). In some cases, people with disabilities are hidden by family members from the wider community, and in extreme cases, children with disabilities are killed to avoid the shame it brings to the family (Baffoe 2013). In addition, disability is also seen as a result of witchcraft, sorcery and ‘juju’ (magical powers) (Baffoe 2013; Naami 2014). For example, some Ghanaians believe that children with epilepsy are filled with demons that sometimes torment and throw them on the ground, when they so wish. Also, children with Down syndrome and CP in Ghana are believed to be children given by the river gods, called ‘nsuoba’, meaning ‘water children’ (Baffoe 2013; Opoku et al. 2015). These beliefs impact on the lives of children with disabilities and their families who are constantly being labelled and stigmatised in societies (Kassah, Kassah & Agbota 2014; Naami 2015; Opoku et al. 2017a). In a society where disability is understood from cultural interpretation (Opoku et al. 2017b), it is necessary to develop an in-depth understanding of the caregiving experiences of primary caregivers of children with CP across the trajectory of diagnoses in Ghana to inform policy directions.

## Theoretical framework

Because of the importance of relationships between people to development, we have situated this study in social capital theory (SCT). This theory has been applied in several social science disciplines to study the relationship between people and agencies in societies (Aldrich & Meyer 2015; Ferfojja, Diaz & Ullman 2018). In this study, we have applied the theory as it pertains to youth studies and community development to interpret the results of the study. Social capital theory refers to individuals’ position within a society that impacts on their lives (Ferfojja et al. 2018). This covers the services available to people as a result of networks or acquaintance with people in the society. Both formal and informal networks impact on the lives of individuals in the society. An informal network relates to the bridge between people and close units such as family and members of the community (Aldrich & Meyer 2015; Sime & Fox 2015). Here, the attitudes and perception towards an individual impacts on their ability to access social capital such as education and resources for development. A formal network refers to the bond between people and structures in the society such as education, jobs and other essential services (Aldrich & Meyer 2015).

In this study, the researchers are interested in the relationship between caregivers of children with CP and networks in society, both informal and formal. The informal network refers to the experience of caregivers in terms of their relationship with other people in the society. The formal network is linked to the experience of caregivers in accessing essential services for their children.

## Primary caregivers of children with cerebral palsy

Children with CP have been reported to require a high level of physical care and emotional support from primary caregivers. This suggests that primary caregivers may be in need of education, training and being exposed to support services available to enhance the well-being of children with CP. However, it seems parents with children with CP in low-income countries such as Ghana have limited information about rehabilitation, training and feeding of their children (Polack et al. 2018; Zuurmond et al. 2019). Indeed, a study by Olawale, Deih and Yaadar (2013) found that primary caregivers indicated that they required adequate knowledge of CP to help them cope well with the demands of taking care of the children with CP. As a result of limited information on support services for children with CP, caregivers tend to rely on informal support from relatives and close friends in society (Zuurmond et al. 2018). However, in the midst of negativity towards children with disabilities and caregivers, such assistance maybe unreliable. This necessitates the need for more information on experiences of primary caregivers to rely on evidence to impact policies.

Elsewhere, studies have reported the experiences of primary caregivers of children with CP. It is apparent that in many advanced societies, primary caregivers of children with CP are much involved in raising their children and develop in-depth understanding about their children’s diagnosis and needs (Whittingham et al. 2013). Because of their level of awareness, they may be able to advocate for their children and support their inclusion in societies. Also, primary caregivers are happy when appreciated for their extra roles of assisting their children or when their children with CP are accepted and lived independently (Wiart et al. 2010). In the effort towards creating an inclusive society, caregivers are satisfied when their children with CP are recognised as equal members of society.

Despite the involvement and positive experience of some caregivers raising their children with CP, extant literature has reported barriers they faced in raising their children with CP (Garip et al. 2017; Khayatzadeh et al. 2013; Kuo & Geraci 2012; Tseng et al. 2016). For example, primary caregivers of children with CP had lower incomes (Whittingham et al. 2011), lower quality of life (Khayatzadeh et al. 2013; Terra et al. 2011) and poorer health outcome (Garip et al. 2017) than that of the general population. In addition, it has been reported that primary caregivers were predisposed to psychological stress and at risk of psychiatric disorders (Majnemer et al. 2012; Whittingham et al. 2013). The stress levels have been reported to be related to the levels of support needed by the children and severity of their disability (Garip et al. 2017); Tseng et al. 2016). However, it seems little has been done in terms of documenting the experiences of primary caregivers across the diagnosis trajectory. Given the need to create an inclusive society and develop social capital of all persons, coupled with lack of social support services and paucity of contemporary literature to engage policymakers in Ghana, the authors sought to explore the experiences of primary caregivers across the trajectory of the diagnosis (before, during and after) of CP in the setting of a tertiary hospital. The outcomes of this study will have policy implications with regard to improving the well-being of primary caregivers and children with CP.

## Methods

### Participants

Participants for this study were primary caregivers of children with CP who were purposively recruited from the Out-Patient Department of Child Health at the Korle Bu Teaching Hospital during the neurological clinic consultations on Mondays. The hospital is the largest tertiary health facility in Ghana that provides specialist care to children with CP. Participants were included based on the following criteria: (1) she or he is the primary caregiver of a child with CP between the ages of 1 and 15 years; (2) his or her child has been formally assessed by a multidisciplinary team and confirmed to have CP and (3) she or he is capable and willing to give consent to take part in this study.

Table 1 summarises the characteristics of participants who took part in this study. Overall, 40 primary caregivers took part in the study. Of these participants, 87% were female and 13% were male. The age range of participants was between 22 and 57 years and the majority of participants (60%; *n* = 24) were between 30 and 40 years of age. The majority, 40% (*n* = 16), of the participants had attained a Junior High School education qualification. A total of 30 (75%) of the 40 participants were married at the time of the interview and 50% (*n* = 20) of participants were self-employed such as traders.

**TABLE 1:** Distribution of demographic characteristics of participants.

Category	Characteristics	Frequency (*N* = 40)	Percentage
Gender	Male	5	13
	Female	35	87
Age (year)	20–30	5	13
	30–40	24	60
	40–50	8	20
	50–60	3	7
Level of education	Tertiary	7	18
	Diploma or certificate	9	22
	Senior High School	8	20
	Junior High School	16	40
Occupation	Formal employment	10	25
	Self-employed	20	50
	Unemployed	10	25
Marital status	Married	30	75
	Divorced	1	3
	Separated	3	7
	Widow	1	3
	Single	5	13
Relationship of caregiver to child	Mother	32	80
	Father	5	13
	Grandmother	3	7

### Instrument

Descriptive design was adopted for this study to understand the experiences of primary caregivers of children with CP before, during and after diagnoses. The researchers’ primary goal is to report the subjective views of a population and discuss its implication to policy for policy and practices (Babbie 2011). The primary caregivers of children with CP are aware of their daily life experiences and as such are able to give insight into their world. To listen to primary caregivers, we developed a semi-structured interview guide, based on study objectives and review of literature. The interview guide covered the three broad objectives of the current study within the diagnostic trajectory: the experiences of primary caregivers of children with CP before, during and after diagnosis (see Appendix 1).

### Procedure

The study and its protocols were approved by the university, hospital and the Head of Department of Child Health. Clinical records of prospective participants were reviewed to ascertain proof of diagnosis and selection. The nurses’ pulled-out contacts of primary caregivers were obtained from the clinic database. They contacted them on behalf of the researchers. Information about those who agreed to participate in this study were given to the research team. Sixty-three primary caregivers met the inclusion criteria and were recruited. However, after consenting to participate, 23 primary caregivers either rescinded or did not show up on the agreed dates of interviews. Forty participants were recruited for this study.

The first author conducted the interviews from November 2013 to April 2014. On the days of the interviews, after participants and their children were treated by health professionals, they were ushered into an office where the interviews were conducted. Before the interviews, nurses at the facility counselled participants. During the counselling, they were informed of the nature of the study, some of the questions to expect and the relevance of the study. They were informed that sharing their experience could impact on their psychological well-being. However, they were told that participating in this study and discussing their experiences are a way of sharing vital information for national development.

At this stage, participants were left in the room with the first author. Each participant signed a written consent form before they were interviewed. The study objectives were discussed with potential participants, and possible risks and benefits of the study were shared with each of them. Participants were told that they had every right to withdraw from the study without any negative consequences. They were assured that their identity will be kept confidential throughout reporting of the study as no identifying information will be published. Almost all the interviews were conducted in Twi, which is the language spoken by many people in Ghana. The interviews lasted between 30 min and 1 h, and were recorded with a voice recorder.

### Data analysis

The interviews were transcribed verbatim by the first author who is proficient in the native language (Twi) and English. While playing the audio, she translated and entered the responses of participants in English to a Microsoft Word document. After she transcribed the first five interviews, the third and sixth authors, who are also proficient in both languages, translated and transcribed the same interviews. The three authors compared their transcriptions and realised that they had 85% agreement; however, they reached agreement on some areas they disagreed. Afterwards, the first author continued to transcribe the remaining 35 interviews.

Four of the authors met to discuss the transcribed data. The first author briefed the remaining authors about trends that emerged in the transcriptions. These were issues categorised under the three components of the interview guide (experiences before, during and after diagnosis). Subsequently, phone calls were placed to all participants by authors 1 and 4 to discuss key themes that emerged in the interviews, and to confirm if their views had been captured appropriately and to gain consent to use data in this study. All the participants gave the research team permission to use their data to write this article.

We performed thematic analysis following steps outlined by Braun and Clarke (2014). The steps followed were reading, coding and developing framework, categorisation and mapping, tabulation under themes, extraction of data and writing of draft results. It is important to point out here that the components of the research questions were used as a priori codes. In analysing the data, firstly, the data were read by the research team individually to familiarise themselves with the content. At this stage, a meeting was held to discuss phrases to use as codes. Following this, all the authors coded the data and developed separate coding frameworks. The authors met to discuss the coding frameworks and ascertain if they all assigned the same descriptors to interviews. The authors compared the coding framework, solicited suggestions and reached consensus on a common coding framework. The similarities and differences between coders were mapped and categorised under sub-themes. The sub-themes were then tabulated under the a priori themes (experiences before, during and after diagnosis). The sub-themes and associated codes were transferred onto a new Microsoft Word document and texts associated with them were extracted to the document. The third author wrote the story line and the first draft of results was shared among the authors. All the authors made suggestions that were incorporated in the results reported in this study.

### Ethical considerations

The study and its protocols were approved by the Committee on Human Research, Publications and Ethics (CHRPE/AP/304/13), School of Medical Sciences, Kwame Nkrumah University of Science and Technology, Ghana.

## Results

Participants reported barriers they encountered at each stage of the diagnostic process. The experiences of participants are presented under three sub-themes, namely primary caregivers of children with CP’s experiences before, during and after diagnosis.

### Experiences before diagnosis

Results presented under this sub-theme reflect complications and factors that according to primary caregivers contributed to giving birth to a child with CP. Other remaining parts of this section highlight perception and superstitious beliefs participants have heard about children with CP.

Of the 40 participants, eight indicated that they experienced complications during the birth process. Two of them had induced labour and were allegedly inadequately managed. One participant allegedly experienced a cord complication but was made to deliver vaginally which also resulted in birth asphyxia (lack of oxygen for infants). Another participant, who is a health worker, asked to be sedated to ease the labour pains. She became weak because of the sedation and in the process was unable to deliver the baby spontaneously. Eventually, she was assisted by colleagues to deliver vaginally, but the baby sustained a brain injury. The last three participants reported their children convulsed later after birth before their first birthday (Table 2).

**TABLE 2:** Summary of thematic analysis.

Thematic themes	Sub-themes	Codes
Experiences before diagnosis	Complications at birth	Induced labour, convulsion, jaundice at birth, no cry at birth
Experiences during diagnosis	Encounter with health professionals	Regular postnatal, health professionals downplaying concerns, limited time to see professionals
	Superstitious beliefs	Cultural practices, cultural interpretations, pressure to exterminate child, forced out of homes, marital problems
Experiences after diagnosis	Experience raising children with CP	Emotional reactions to CP, difficulty raising children with CP, depression raising children, education and acceptance
	Neglect in families and communities	Neglect by partner and family, negative impact on social life, negative attitude towards participants
	Experience of hardship	Financial impacts, quitting jobs, cost of medications, lack of assistive devices
	Inaccessibility of hospitals	Lack of specialist care, long distance to seek healthcare, inaccessible facilities, poor transport systems, long wait at facility
	Inaccessible education services	Limited opportunities, denial of admission in regular school, limited facility to cater for children, lack of resources

CP, cerebral palsy.

Sixteen (40%) primary caregivers reported their infants had jaundice at birth. The following are vivid experiences of two of the participants:

‘When I gave birth to him, after three days he become yellow and we sent him to the hospital and they told us to expose him to the sun, but still he become more yellow and we sent him back. He was admitted and put in an incubator.’ (Mother A, female, 32 years old)

‘Her mother was a student when she got pregnant with baby P, when she gave birth to her, she became very sick after 5 days. The doctor said it was jaundice, so they took her blood and replaced it with another and since then we have always been coming to the hospital… We virtually live in the hospital.’ (Grandmother A1, female, 58 years old)

Out of the 40 participants, eight also reported their babies did not cry at birth and two recounted as follows:

‘At birth, she didn’t cry. The nurses took her away for about three (3) hours and later told me she had to be kept in the incubator. Upon my enquiry, the nurses told me she didn’t have the energy to breathe. I could only pray for her survival then. I heard her first cry when she was a week old, but it didn’t sound like the way other babies cry.’ (Mother U, female, 41 years old)

‘Baby J, was very adorable at birth. She didn’t cry, the nurse pinched her feet several times to stimulate her to cry but it didn’t happen. Eventually, she made some sounds as if she was in pain. I wasn’t sure what was wrong. During weighing, I complained to the nurse, but she asked me to give her some time as the children can be a bit different sometimes.’ (Mother C, female, 25 years old)

### Experiences during diagnosis

This section presents support participants received from health professionals and its effect on their children with CP. It also covers the traditional consequences and initial reaction from family members when their children were diagnosed with CP.

#### Encounter with health professionals

Of 40 participants, 32 said they visited the hospital to understand their children’s condition. This action marked the beginning of a long and difficult journey for primary caregivers as they begun to interact with multiple professionals to obtain a clinical diagnosis for their child’s condition. Primary caregivers felt strongly that health professionals initially did not take their concerns seriously which later complicated their children’s situation.

‘At four months I realised my child wasn’t sitting, I was very worried as his siblings at that age would be sitting with some support and playing on their own. I knew there was something wrong but when I complained at the hospital, all they told me was that it was normal for some children.’ (Mother R, female, 28 years old)

‘I had my first child with this kind of condition. I have been visiting the Neuro clinic. … Unfortunately, the second child came with the same condition and nobody is willing to tell me the cause of this condition. … When I ask the doctors, none can tell me what the actual cause is. It is very a difficult situation for me because I thought this is where I could get the answers to my questions.’ (Mother G, female, 34 years old)

#### Superstitious beliefs

Superstitious beliefs are common attributes in the African culture. Of the 40 participants interviewed, 20 commented they had experienced some of these superstitious attributes that existed within their culture. Five participants reported they had been accused of bringing forth ‘spirit children’, some referred to the children as ‘nsuoba’ (river child), whereas others were blamed for bringing taboos to the families of their husbands. Participants recounted that they were pressured to kill their children with CP so as to avert future calamities. Some participants commented as follows:

‘My father called me and informed me that there was some kind of ritual that had to be done for me. After some time, my father called me again, this time with my husband and insisted that the ritual should be done and that it was a taboo to keep such a child in the family; the ritual was long overdue….my husband helped me pack my belongings and sneaked me to the station to pick a bus to Accra with our two children.’ (Mother I, female, 30 years old)

‘Six months after giving birth, I realised he was not sitting . … My mother in-law also realised this. It was after this event that her attitude towards me changed …. she distanced herself and informed other family members about the condition .… She advised that we consulted the elders of the town to ‘escort the child’ and that it was best we returned him to where he came from .… I advised myself when I could not bear the pressure anymore to relocate.’ (Mother F, female, 33 years old)

One participant recounted how she was thrown out of her husband’s family house after realising her child could not sit at 6 months, could not keep his head straight after 8 months and kept drooling at age 1 (Mother E, 25 years old). Another mother reported that her husband left their house after their child was born because of pressure from his family (Mother H, female, 32 years old).

### Experiences after diagnosis

This section presents results of experiences encountered by participants after their children were diagnosed with CP. The section explains the experience of participants raising children with CP, relationship between participants and people in the society, effect of child with CP on their lives and accessibility of essential services such as education and healthcare to children with CP.

#### Experience raising children with cerebral palsy

Almost all participants (*n = 36*) in the present study confirmed that they were affected by their children’s diagnosis of CP and they sometimes grieved as if their children did not exist. They expressed varying emotions, namely initial shock and denial; anger and resentment; depression; and eventual acceptance. Initial shock and denial were expressed in the following ways:

‘I accepted it with much ease than my husband did. I think it was because I had more frequent contact with baby K on a daily basis than he did. He almost pretended as if it wasn’t happening. … He thought baby K would grow out of it or that something would change.’ (Mother E, female, 25 years old)

‘I woke up every day hoping to see a change … that she, a child with cerebral palsy would get on her feet again and play with her siblings as she used to. I had a strong believe it was something temporal; she was probably just feeling very weak, making her unable to walk until the doctor advised us to get her a wheelchair and start physiotherapy.’ (Mother M, female, 31 years old)

A few participants also expressed anger and discussed the difficulties they encountered in caring for their children with CP. For example:

‘I don’t know whether to call it anger, these are innocent children. It is difficult to explain. … I just get so frustrated and bitter sometimes. I have to halt my life and take care of him from dawn to dusk. If only his dad would show a little concern; his understanding of caring for a child is just his upkeep monies, that’s all.’ (Mother N, female, 34 years old)

Of the 40 participants, 26 reported feeling depressed. This was in the form of, combination of guilt, sorrow and tearfulness. For example:

‘My eyes are always filled with tears when I remember that day. I somehow believe I’m being punished for my sins. To think of the fact that if I had not taken that injection and went through the normal labour pains, this might not have happened.’ (Mother Q, female, 37 years old)

‘Sister (referring to the interviewer), I am a Christian, but now I doubt my faith; I doubt if there is any God. One child with this condition is already a headache, I have two! What did I do wrong to merit this? I don’t believe in any God because he is not there to see my plight. I walk around always questioning myself, what I did wrong. Now, I’m scared to even think of having any more kids.’ (Mother G, female, 34 years old)

Fifteen out of the 40 participants reported that they were able to attain some level of acceptance once they adjusted their expectations regarding their children’s capabilities and what the future held for them. Participants reported that their concerns and anxiety lessened once the children were diagnosed and were in a position to seek appropriate treatment and educational programmes suitable to their children’s unique needs. Participants’ sense of relief was reflected in the following statements:

‘After the doctors confirmed that a part of the brain had been damaged resulting in her loss of speech and movement, I took it upon myself to research about the condition and ask parents with children of similar condition. … Now, I know, this is the turn my life has taken, I am willing to help her go through life with the help of God.’ (Mother M, 31 years old)

‘With “D”, my wife and I got some sense of relief when the professionals finally explained the repercussion of the condition. I am able to help with “D” in the house since I’m on retirement. My wife’s hands are already full with the two other children.’ (Father B1, male, 27 years old)

#### Neglect in families and communities

Throughout the accounts of the 40 primary caregivers interviewed, 16, mostly mothers, indicated that they were fully responsible for taking care of their children. While fathers indicated their willingness to support the care of their children, mothers said that they felt neglected by their spouses and other family members. One mother reported that though she was staying with the husband and still married, the husband showed very little concern in the condition of their child. Another mother reported that the husband felt too embarrassed to show their child in public and had ordered her not to send the child out into the public.

‘My husband’s family do not visit anymore, I’m not invited to family gatherings anymore because they do not accept “K” as one of their own and if I could give birth to such a child, then I’m not worth joining the family.’ (Mother H, female, 32 years)

All participants reported that having a child with CP had affected their social life in one way or an other. A mother shared her ordeal with her child when she stepped out. She reported that:

‘Going out with “P” is a whole mission now … the community in which we live shun children with such condition. The moment you step out with him and his clumsy movements, all eyes turn to look at you. We cannot attend functions anymore at church, it is quieter and easier caring for him at home than to experience the ‘rolling eyes’ when we go out.’ (Mother X, female, 32 years old)

‘The Ghanaian society and for that matter Africans as we are, we depend on each other, but now I cannot honour invitations to funerals and other important events in people’s lives after having my child. It got to a time, I realised I was no longer getting the invitations. … the sad part is no one will actually check on you to know why and sympathize with whatever condition you may be going through.’ (Mother S, female, 41 years old)

#### Experience of hardship

When asked how caring for their children affected them financially, all participants complained that it had a huge toll on them. While mothers were concerned about their inability to work, fathers were concerned about high cost of medicines and related equipment. Specifically, 18 mothers reported they had to stop working or quit their jobs to take care of their children with CP. They felt that they were unable to go out to work as their children required constant care and they felt they were unable to leave the child with anyone else while they went out to work. Two mothers recounted their ordeal as follows:

‘I have to take him everywhere I go. It is very difficult to get someone to help look after him, they complain that he cannot express himself and that makes it very difficult for them to understand when he wants to go to toilet or when he is hungry or unhappy about something.’ (Mother T, female, 38 years old)

‘I just had to quit my job to look after baby “J”. My mother who was helping me after his delivery was beginning to get tired of taking care of him. … It was at that point that I realized I had to make a decision to quit my job to take care of him.’ (Mother C, female, 25 years old)

All the men who took part in this study expressed difficulty in procuring the prescribed medication and equipment for their children that would help to improve their functioning. They complained about the cost of medication for their children that were not covered by the National Health Insurance Scheme and they had to purchase it themselves. This according to them is a drain on their finances.

‘There are about three medicines the doctors always prescribe for him, but none is covered by the health insurance. I have to buy these medicines every month. The hospitals do not also provide assistive devices to help them move … I will have to go and get it myself, but I cannot afford it. As I talk to you, I had to go to the roadside to find a taxi to come and pick him from the house to the hospital.’ (Father B2, male, 41 years old)

‘I must admit it is a very difficult situation the family finds itself, especially when it comes to finances. Being the father, this major task is on me as the mother is unable to work because she has to take care of our sweet daughter. … This expense is aside the medications she has to take. … I have spent all my savings on our daughter’s care, I have sold my car and now taking public transport just to be able to afford the care for her.’ (Father B3, male, 52 years old)

Generally, participants with children above 5 years, who could not walk, complained about the non-availability and cost of equipment, especially wheelchairs for their children. Some primary caregivers with toddlers also mentioned the non-availability and cost of standing frames for their children as major challenges.

#### Inaccessibility of hospital

The inaccessibility of specialist clinics was of unanimous concern among all primary caregivers as some had to travel from other regions to access healthcare in the Greater Accra Region where this tertiary care is available. Only three primary caregivers lived in close proximity (about 2 km) to the study site. Interestingly, while those who lived close complained about frustrations they go through at the hospital, those who lived outside the city were concerned about transportation to the facility. Three mothers who participated in the study said they felt that the hospital was inaccessible. One mother who lived 190 km away reported as follows:

‘The district general hospitals do not have this kind of clinic, so we have no option than to travel the distance to access it and it becomes costlier when we have to charter a vehicle because most public vehicles will not let you board their vehicle when you have a child with such condition.’ (Mother B, female, 34 years old)

Another mother who lived about 12 km from the main tarred road where public transport could be accessed said that ‘I have to pay people to carry him to the roadside before we can get a car to the hospital. I repeat the same when we return from the hospital’. Another mother shared the frustrations she goes through when they finally get to the hospital:

‘This is the only hospital in the capital that runs a neurodevelopment clinic. After hustling with transportation to get to the hospital, we have to queue and get numbers to stay in the queue waiting to see the doctor who comes at 2pm in the afternoon. Meanwhile, we have to get here early in the morning to get a good position in the queue in order to see the doctor early.’ (Mother V, female, 35 years old)

#### Inaccessible education services

Almost all participants discussed the difficulties encountered as they searched for suitable day care and educational facilities for their children with CP. Twenty-four (60%) primary caregivers were still searching for educational facilities that could admit their children. Many reported that mainstream schools were unable to meet the special educational needs of their children as they were constantly denied admission. For example, two mothers reported that they had been to mainstream schools and turned away because they did not have facilities and personnel to take care of such children. Interestingly, special schools were unable to admit their children because they did not have the capacity to support their children. Some primary caregivers shared their experiences as follows:

‘When “P” finally started walking, my greatest joy was that he could finally attend school just as his peers, but that dream was far-fetched as I was turned away by many public schools. They told me they did not have the personnel to take care of him. One principal told me ‘such children disturb the class when the children are learning.’ (Mother X, female, 32 years old)

‘Someone told me to try the special schools, I was very hopeful that at least, mingling with his kind was better than none but again I was turned away by two special schools. They complained they were not in the position to give such intensive care for children with cerebral palsy and if only I could get him a personal assistant, then they could consider admitting him.’ (Mother W, female, 37 years old)

## Discussion

In this study, the experiences of caregivers raising children with CP were explored using SCT as framework. It thus seeks to increase the knowledge base about what it means to have and live with a child with CP in the Ghanaian context. At each stage of the diagnostic trajectory, it was found that there were barriers encountered by caregivers. For instance, the results show that during diagnosis some primary caregivers were being pressured to exterminate their children with CP. This was made known through constant pressure by in-laws or parents of primary caregivers to neutralise these children. This finding is partly consistent with other studies conducted in Ghana that reported rejection of children with disabilities and primary caregivers in societies (Kassah et al. 2014, 2018; Naami 2015; Opoku et al. 2017a, 2017b). This means that having a child with CP affects the relationship between individuals and people within the society. This could adversely impact on socialisation of caregivers and their children with CP. It is apparent that informal network may not support caregivers to nurture the potential of their children. In particular, the decision of primary caregivers to keep their children meant that they had disrespected their elders and that is considered to be a major offence in Ghanaian societies, although this was not revealed in this study. It seems society is yet to accept children with CP as equal members of the society, but until then primary caregivers and their children may need to be marginalised and excluded from building bridges with people in the society.

The relationship between individuals and other groups in the society impacts on development (Ferfolja, Diaz & Ullman 2018). The ability of individuals to obtain useful information and assistance could enhance their development. However, in this study many participants were unhappy about services provided to them by health professionals. Notably, almost all participants indicated that they visited health facilities to understand their children’s condition, but they were dissatisfied with the explanation given to them by health professionals. This finding partially corroborates previous studies that found that primary caregivers of children with CP were unable to access health services, lacked information and were dissatisfied with the support they received from health professionals (Olawale et al. 2013; Polack et al. 2018; Zuurmond et al. 2019). It is likely that the health professionals did not have the necessary resources and skills to provide effective care and counselling to the children with CP and their caregivers. Many studies have reported on limited infrastructure at health facilities to promote the needs of individuals with disabilities in Ghana (see Badu, Agyei-Baffour & Opoku 2016b; Badu, Opoku & Appiah 2016a; Senayah et al. 2019). Apparently, the health professionals were helpless to provide participants with useful information to avoid having and caring for children with CP. It is possible that children with CP will be denied effective participation in social services in societies because information about their upbringing was unavailable to their caregivers.

The position of individuals in society could affect accessibility of services (Ferfolja et al. 2018). In this study, many primary caregivers recounted that they experienced financial difficulties after their children were diagnosed with CP. Participants discussed that they had to cover the cost of medical and transportation expenses which put strain on their finances. This finding corroborates the results of previous studies that found that primary caregivers of children with CP suffered acute financial difficulties that affected their psychological well-being (Dogbe et al. 2019; Khayatzadeh et al. 2013; Whittingham et al. 2011). This finding is unsurprising because there are limited social support systems available to parents and their children with disabilities (Opoku et al. 2019). Compounding this situation is negative attitudes people in society have towards people with disabilities in their families. It is apparent that participants had to shoulder all the burden of caregiving and provide for their children with CP. It is possible participants may have other responsibilities such as raising typically developing peers that might interfere with their ability to support children with CP. In an era where much advocacy is focused on achieving an inclusive society, participants may not be able to support their children with CP to participate in basic services in the society. This could affect their self-worth and capacity to live independently.

The interaction between individuals and essential services is instrumental in developing social capital (Ferfolja et al. 2018). This includes barrier-free access to education, which has been found to be an important agency in societies. In this regard, many participants recounted their experiences about accessibility of schools to children with CP. This frustrating narrative resonated loudly through the discussion with the participants who shared that neither regular nor special schools were prepared to admit their children. In Ghana, apart from the hospitals, the only established places where there are specialist services are special schools (Kassah et al. 2018). It is believed that both regular and special schools have specialised staff who could render services in terms of behaviour management and socialisation (Kassah et al. 2018). However, this study found the contrary. These facilities were discussed by participants as poorly resourced and lacking appropriate capacities to educate children with CP. In the absence of any standardised external support, it is unsurprising that the primary caregivers had no other choice, but to keep their children at home and play the role of teachers, further perpetuating the cycle of poor education, poverty, marginalisation and exclusion. This is the never-ending trend of the cultural norms in the Ghanaian society that has minimal room for disability and its outcomes (Agyei-Okyere et al. 2019; Baffoe 2013; Naami 2014; Opoku et al. 2018). It is therefore likely that participants would be over-stretched with their coping strategies, while their children grow without basic skill that will enable them to integrate and become independent adults.

## Study limitations and future research direction

It is important to state that there are a number of limitations that make it impossible to generalise this study. The study was limited in scope as participants were recruited from the main referral hospital for CP in Ghana. Also, the study focused on the experiences of parents with CP which might be different from parents with children with other disabilities. Future research should use quantitative methods to expand this study and compare the experiences of primary caregivers of children with different types of disability to get a clear picture of parental experiences. Nevertheless, this study has provided a snapshot of the experiences of parents with children with CP across their diagnosis.

## Conclusion and policy implications

Social capital theory was used to underpin this study to explore the experiences of primary caregivers of children with CP before, during and after the diagnostic process. Although the relationship between people in society and agencies is critical to human development (Ferfolja et al. 2018), it seems participants might be unable to support children with CP. The results show that at every stage in the diagnosis of a child with CP, participants said they encountered barriers that have repercussion on caregiving experiences. For instance, it emerged that participants suffered emotional distress shortly after giving birth to children with CP. Particularly, some participants were confronted with doubts and confusion in trying to understand the emerging health problems and/or developmental abnormalities in their children. Also, some participants were confronted with superstitious beliefs and were persuaded to act as directed by the heads of their families and/or leaders in the communities in which they lived. In addition, many participants encountered financial problems as they were confronted with purchasing medicine and transporting their children to health facilities. Similarly, primary caregivers of children with CP face further challenges as they search for suitable day care and education for their children. As it stands now, achieving an inclusive society where participants’ children with CP could develop their talents may be far-fetched looking at the experiences reported in this study. These findings seem to underscore the urgency for structures to be put in place to improve the lives of participants and their children with CP.

The findings of this study have implications for policymaking in Ghana. The findings seem to underscore the urgency for social and economic reforms on multi-sectorial platforms of government and non-governmental agencies. Firstly, there is the need for health policymakers to intensify disability awareness campaigns and promote the well-being of primary caregivers. Secondly, there is the need for the establishment of support groups by social workers in communities and hospitals for primary caregivers of children with CP. Thirdly, government and private individuals should resource and promote the educational needs of children with CP in Ghana that can provide daily living skills to them. Similarly, there is the need for government and other stakeholders to resource hospitals to promote accessible health services to primary caregivers and their children with CP.

## Appendix 1

Interview guide schedule for participants.

Researcher introduces self and summarises the aim of the study. Could you tell me more about yourself?

### Experiences before diagnosis

1. How was your pregnancy experience?
2. How was your experience during childbirth?

### Experiences during the diagnostic process

1. What prompted you to seek help for [child’s name]? Tell me about this time in your family’s life.
2. Who first mentioned cerebral palsy? What sense did you make out of such diagnosis/what did that diagnosis mean to you?
3. What was particularly difficult during this time?
4. What was helpful?
5. How did you feel during this stage?
6. What did you do/feel like doing after you were told? What thoughts and concerns ran through your mind as you tried to understand your child’s condition?

### Post-diagnostic experiences

1. Tell me about the general running of the household after [child’s name] was diagnosed with cerebral palsy. How is that different now? How did you manage daily tasks such as feeding, meals, bath time, using the toilet, bedtime, fun activities and so on?
2. What difficulties did you encounter? What helped?
3. How did you cope with all the demands on your time and energy? What or who was the most helpful in coping with the demands of raising your child with cerebral palsy? Who did you turn to for help?
4. What formal support systems and helpful organisations are available to you? What informal support networks have helped you through tough times? How did you experience the efficacy of these resources?
5. What do you still need? How can the services available to caregivers of children with cerebral palsy be improved?
6. What are your thoughts about [child’s name] future? What are your main concerns about him or her? What gives you hope?
